# Disordered Eating Behaviors Related to Food Addiction/Eating Addiction in Inpatients with Obesity and the General Population: The Italian Version of the Addiction-like Eating Behaviors Scale (AEBS-IT)

**DOI:** 10.3390/nu15010104

**Published:** 2022-12-25

**Authors:** Alessandro Alberto Rossi, Stefania Mannarini, Gianluca Castelnuovo, Giada Pietrabissa

**Affiliations:** 1 Department of Philosophy, Sociology, Education, and Applied Psychology, Section of Applied Psychology, University of Padova, 35131 Padova, Italy; 2 Interdepartmental Center for Family Research, University of Padova, 35131 Padova, Italy; 3 Psychology Research Laboratory, Ospedale San Giuseppe, IRCCS Istituto Auxologico Italiano, 28824 Verbania, Italy; 4 Department of Psychology, Catholic University of Milan, 20123 Milan, Italy

**Keywords:** eating behaviors, food addiction, binge eating, obesity, eating disorders, eating behaviors

## Abstract

Purpose. The purpose of this research is to test the psychometric properties and factorial structure of the Addiction-like Eating Behaviors Scale (AEBS) in an Italian sample of adults with severe obesity seeking treatment for weight reduction and the general population, and to examine the measurement invariance of the tool by comparing a clinical and a nonclinical sample. Methods. A confirmatory factor analysis (CFA) was initially conducted to test the factorial structure of the Italian version of the AEBS (AEBS-IT) on a total of 953 participants. Following this, the measurement invariance and psychometric properties of the tool AEBS-IT were assessed on both inpatients with severe obesity (*n* = 502) and individuals from the general population (*n* = 451). Reliability and convergent validity analysis were also run. Results. CFA revealed a bi-factor structure for the AEBS-IT, which also showed good reliability and positive correlations with food addiction (through the mYFAS2.0 symptom count), binge-eating symptoms, compulsive eating behavior, and dysfunctional eating patterns and the individuals’ body mass index (BMI). Moreover, the tool was invariant across populations. Conclusion. This study provided evidence that the AEBS-IT is a valid and reliable measure of FA in both clinical and nonclinical samples.

## 1. Introduction

Obesity is on the rise, and it is predicted to rise to nearly half the global population by 2030. Recent statistics suggested that 50% of men and 55% of women, globally, are overweight or obese [1]. This is problematic given the adverse health and social factors associated with obesity, such as decreased quality of life and increased morbidity and mortality [2,3].

One of the central causes of obesity is an energy imbalance of calories consumed and expended [1]. However, the onset and development of obesity are complex and include several factors such as psychological, sociological, biological, evolutionary, economic, and institutional factors [4,5]. Obesity is a preventable and treatable condition, with strategies frequently focusing on reducing sedentary lifestyles and improving dietary intake, however, long-term success is limited, and relapse is frequent [6]. Therefore—to inform clinical interventions and contribute to health prevention strategies—it is important to concentrate on other explanations that may contribute to the behavioral phenomenon of food overconsumption. The last two decades have been characterized by increased interest in the construct of Food Addiction (FA), a substance-use disorder (SUD) characterized by excessive overeating of hyper-palatable and high-energy-density foods—including sugars and fats—often comprising patterns of loss of control and intense food cravings that have the potential to generate dependence [7,8].

Consistent with this idea, according to criteria for SUD, Gearhardt et al. (2009) [9,10] developed the first measure of FA, the Yale Food Addiction Scale (YFAS) and its subsequent versions (i.e., YFAS2.0 and mYFAS2.0), a self-reported questionnaire adapted from the Diagnostic Statistical Manual (DSM)-IV-TR [11] and DSM-5 [12,13,14,15,16]. Since its publication, the YFAS and its subsequent versions have been used as the instrument of choice for measuring FA—contributing to stimulating research and establishing preliminary findings in the field [17,18,19].

However, some authors have proposed that the criteria for SUDs should not be applied to food—suggesting several fundamental differences between drugs and food [20,21,22]. For instance, they consider addiction symptoms, such as tolerance, withdrawal syndrome, interference in daily activities, or use in physically hazardous situations, to not be applicable to food consumption and to be more specific to alcohol and drugs [7,23]. Indeed, contrary to alcohol and drugs, food is vital, and considering it an addictive substance should be a matter of concern. Consequently, Hebebrand and colleagues (2014) proposed that FA should be restructured as a behavioral addiction—proposing the denomination of “eating addiction” (EA) [7].

Indeed, behavioral addictions are related to a substantial excitation generated by pleasurable stimuli and a low capacity for inhibitory control, favoring the repeated engagement in that behavior, even in the face of eventual harm [24].

Consistent with this idea, the Addiction-like Eating Behaviour Scale (AEBS) was developed as an alternative to a substance-based conceptualization of FA [21]. Instead of relying upon the SUD model, this 15-item scale aims to quantify the core cognitive and behavioral processes underpinning addictive patterns of the over-consumption of food via two subscales (“appetitive drive”, 9 items, and “low dietary control”, 6 items) consistent with the dual-process theories of motivation related to FA/EA: (1) an increased responsivity to reward-related cues coupled with (2) a diminished ability to exert inhibitory control [25].

In its validation study, the AEBS showed good convergent validity with binge eating, emotional eating, and disordered eating as well as good divergent validity with alcohol addiction and a measure of behavioral inhibition and activation.

The instrument also explained body mass index (BMI) variances above that were accounted for by both the YFAS and the Binge Eating Scale (BES). These results were successfully replicated in a clinical and community sample of French–Canadian participants [26], and the AEBS also demonstrated good reliability and validity in evaluating addictive eating behaviors among the general population in Brazil [27].

Since the AEBS is not yet available for use by Italian clinicians and researchers, the purpose of the present study is twofold. First, it aims to examine the factorial structure and the psychometric proprieties of the Italian version of the AEBS (AEBS-IT) within a clinical sample of adults with severe obesity and within the general population. The second aim is to test the measurement invariance of the tool between clinical and nonclinical samples.

## 2. Methods and Materials

A cross-sectional research design was employed to investigate the factorial structure of the AEBS-IT and its psychometric properties across a population of adults with severe obesity and a community sample.

### 2.1. Translation and Cultural Adaptation

International guidelines were followed [28,29]. First, the AEBS-IT was independently translated from its original English version into Italian by two expert clinical psychologists. Then, to guarantee uniformity between versions, a back-translation was performed by an independent translator. The final version of the AEBS-IT (Appendix A) was administered to a sample of 20 individuals (10 inpatients with severe obesity and 10 individuals recruited from the general population) to evaluate the items’ comprehensibility. No additional adjustments were made.

### 2.2. Sample Size Determination

The sample size was decided a priori using the “*n:q* criterion” [30,31,32], thus the ratio between participants (*n*) and the number of (free) model parameters to be estimated (*q*). A ratio of 5 subjects per parameter (5:1; *n*_minimum_ = 450) was guaranteed [30,31,32,33,34,35,36,37].

### 2.3. Procedure

A survey containing a socio-demographic report form, the AEBS-IT, the modified version of the Yale Food Addiction Scale 2.0 (mYFAS 2.0), the Binge Eating Scale (BES), the Measure of Eating Compulsivity 10—Italian version (MEC10-IT), and the Dutch Eating Behavioral questionnaire (DEBQ) was administered to each participant.

Individuals with severe obesity were enrolled within the first week of a one-month weight reduction and rehabilitation program at the IRCCS Istituto Auxologico Italiano San Giuseppe Hospital, Verbania (Italy). In line with previous studies [15,38], participants from the general population were randomly enrolled in Milan and Padua using the snowball sampling technique, personal invitations, and advertisements placed around the university and in cafés and libraries.

This study was approved by the Ethics Committee of the IRCCS Istituto Auxologico Italiano (protocol no 2020_02_18_04).

### 2.4. Participants

An initial sample of 1000 participants was enrolled; however, 47 surveys were omitted due to missing data/answers. Therefore, the final sample comprised 953 participants: 502 (52.7%) inpatients with severe obesity and 451 (47.3%) individuals from the general population.

The sample of inpatients with severe obesity was composed of 218 males (43.4%) and 284 females (56.6%) aged between 18 and 75 years (mean = 52.51, SD = 12.143) and with a BMI that ranged from 35.65 to 83.21 Kg/m^2^ (mean = 43.457, SD = 6.882).

The community sample was, instead, composed of 69 males (15.3%) and 382 females (84.7%) aged between 18 and 70 years (mean = 28.05, SD = 9.595) and with a BMI ranging from 17.30 to 43.44 Kg/m^2^ (mean = 22.389, SD = 4.041).

The following inclusion criteria were applied: (A) being a native Italian speaker; (B) being ≥ 18 years old; and (for individuals with obesity) (C) having a BMI higher or equal to 35 Kg/m^2^ (BMI ≥ 35). Exclusion criteria were: (D) illiteracy; (E) inability to complete the assessment due to vision and/or cognitive impairments; (F) not providing answers to all items; and (G) denial of informed consent. All participants signed a document providing written and informed consent to participate in the study.

### 2.5. Measures

Demographic (age and gender) and clinical parameters (weight and height—used to calculate the individuals’ BMI) were collected, and the Italian version of the following self-report questionnaires were administered:

#### 2.5.1. The Addiction-Like Eating Behavioral Scale—Italian Version (AEBS-IT)

The AEBS-IT [21] is a 15-item questionnaire used to assess eating-related behavioral addiction. Items are answered on a 5-point Likert Scale ranging from 1 (strongly disagree or never) to 5 (strongly agree or always). The AEBS-IT is divided into two subscales: “appetite drive” includes nine items for a possible score ranging from 9 to 45 and “low dietary control” includes six items for a possible score ranging from 6 to 30.

All items can be summed to obtain a global score ranging from 15 to 75. A higher score represents more frequent and severe addictive-like eating behaviors. The AEBS-IT has been shown to be a valid and reliable tool for quantifying the behavioral features of an ‘eating addiction’ and it is correlated positively with other measures of maladaptive eating, such as the YFAS and BMI [21].

#### 2.5.2. The Modified Yale Food Addiction Scale 2.0 (mYFAS2.0)

The mYFAS 2.0 [39,40] is a questionnaire assessing the presence of addictive eating behaviors. It comprises 13 questions evaluated on an 8-point scale that range from 0 (never) to 7 (every day): 11 items reflect the DSM-5 diagnostic criteria for SUD [16] while 2 items assess food-related impairment and distress experienced by the person during the past 12 months. Each item is scored dichotomously (0 = did not meet the criterion, 1 = met the criterion) according to the threshold determined by Gearhardt and collaborators (2017) [40].

The mYFAS 2.0 provides two different scores: the symptom count version (scores ranging from 0 to 11), which sums up the diagnostic criteria that the subject meets; and the diagnostic score, which requires the presence of impairment/distress criteria. To make an FA diagnosis, both scores are used (mild = 2–3 symptoms plus impairment or distress, moderate = 4–5 symptoms plus impairment or distress, severe = 6 or more symptoms plus impairment or distress) [12]. In this study, the mYFAS2.0 showed adequate internal consistency: the KR20 coefficient was 0.857.

#### 2.5.3. The Binge Eating Scale (BES)

The BES [41,42] is a questionnaire measuring binge-eating severity in both community [10] and clinical samples [43]. It comprises 16 items: eight items that describe behavioral manifestations of BED (i.e., eating fast or consuming large amounts of food) and eight items focused on associated feelings and cognitions (i.e., fear of not stopping eating).

Each question has 3–4 separate responses increasing in severity grouped into two subscales (FC—Feelings/Cognitions; and B—Behaviors) and a total score [43]. Assigning to each statement a numerical value ranging from 0 to 3 points (0 = no severity in BED symptoms, 3 = serious problems in terms of BED symptoms), the BES total score ranges from 0 to 46: a score of less than 17 points indicates minimal BE problems; a score between 18 and 26 points indicates moderate BED; and a score of more than 27 points indicates severe BED [44].

Studies carried out in the past decade, mainly with individuals with obesity, have shown that the BES is very sensitive and specific in distinguishing between compulsive and normal eaters [45,46], and a large number of investigations support its reliability and validity as a measure of eating-related pathology [41,42,47,48].

In this study, the BES showed adequate internal consistency. Indeed, Cronbach’s alphas were 0.901, 0.816, and 0.835 for the BES Total scale, the FC scale, and the B scale, respectively.

#### 2.5.4. The Measure of Eating Compulsivity—Italian Version (MEC10-IT)

The MEC10-IT [49,50] is a measure of compulsive eating within the FA framework. It comprises 10 items scored on a 5-point Likert scale with partial semantic autonomy (from 0 = “*Very Untrue*” to 4 = “*Very True*”) and higher scores corresponding to a higher eating compulsivity. In its original validation study [49], the MEC10 displayed adequate reliability (α = 0.946). Similarly, in the present study, the internal consistency of the tool was adequate, with Cronbach’s alpha equal to 0.941.

#### 2.5.5. The Dutch Eating Behavior Questionnaire (DEBQ)

The DEBQ [51,52] is a questionnaire measuring behaviors and attitudes related to ED in both general [10,53] and clinical populations [54]. It comprises 33 items scored on a 5-point Likert scale (ranging from 1 = “*never*” to 5 = “*very often*”) and grouped into three subscales: Restrained Eating (RE), Emotional Eating (EE), and External Eating (ExE), plus a total score. The DEBQ has been shown to be an adequately reliable and valid measure of eating-related pathology with a strong three-factor structure, high internal consistency, and high test-retest reliability after 4 weeks [51,52,53,54].

In this study, Cronbach’s alphas were 0.934, 0.929, 0.969, and 0.835 for the total scale, the RE scale, the EE scale, and the ExE scale, respectively.

#### 2.5.6. Statistical Analyses

Statistical analyses were run with R software [55,56], and the following packages were also used: corrplot [57], lavaan [58,59], plotROC [60], pROC [61], psych [62], psychTools [63], semTools [64], and tidyverse [65]. Graphical representations were performed with the ggplot2 package [66].

The DWLS (diagonal weighted least square) estimator was employed to evaluate the factorial structure of the AEBS-IT [67,68,69,70,71]. Model fit was assessed using (A) the Chi-square statistic (χ^2^), (B) the Root-Mean Square Error of Approximation (RMSEA), (C) the Comparative Fit Index (CFI), and (D) the Standardized Root Mean Residual (SRMR) [67,68,69,71,72]. To evaluate the goodness of fit, the following cut-off criteria were used: (A) statistical non-significance of the χ^2^, (B) an RMSEA lower than 0.08, (C) a CFI higher than 0.95, and (D) an SRMR lower than 0.08 [67,68,69,71,72].

According to its original validation study [21], a first-order model comprising two-factor was specified: 9 items loaded onto the ‘*appetite drive*’ latent factor (from item#1 to item#9) and 6 items filled into the ‘*low dietary control*’ latent factor (from item#10 to item#15). Moreover, considering that the original validation study assumed a ‘*general factor*’ (i.e., total score), two alternative models were additionally tested. First, a single factor model was verified: all the 15 items of the AEBS-IT loaded onto the latent factor ‘*Addiction-like eating behavior*’ (general factor, only). Second, a bi-factor structure was set: 9 items loaded onto the ‘*appetite drive*’ latent factor (from item#1 to item#9), 6 items filled in the ‘*low dietary control*’ latent factor (from item#10 to item#15), and all the 15 items loaded onto an overall factor named ‘*Addiction-like eating behavior*’ (total score).

To select the best factorial structure (namely, the best model), a model comparison analysis was performed. The following set of criteria and cutoffs for model equality were employed: DIFFTEST (equal to Δχ^2^; *p*-value > 0.050) and ΔCFI (<0.010). Considering the great sensibility to the sample size of the χ^2^, the overpass of the ΔCFI cut-off criteria was considered to be evidence of model inadequateness—combined with worse fit indices [67,72,73,74,75].

Measurement invariance (MI) analysis for categorical data was run to test the between-group (inpatients with severe obesity vs. general population) invariance of the factorial structure of the AEBS-IT [76]. According to guidelines [72,76,77], four (nested) models were defined and their model parameters were sequentially constrained to equality: Configural Invariance (Model 1: equal factorial structure); Metric Invariance (Model 2: equal factorial structure and item factor loadings); Scalar Invariance (Model 3: equal factorial structure, item factor loadings, and item thresholds); Means Invariance (Model 4: equal factorial structure, item factor loadings, item thresholds, and latent means) [72,74,75,76,77].

These nested models were sequentially compared. Model calculations were done by means of the aforementioned test differences: DIFFTEST and ΔCFI. Additionally, the same rule-of-thumb for model inadequateness was applied.

The AEBS-IT internal consistency was evaluated with Cronbach’s alpha (α) and McDonald’s omega (ω) [78,79,80].

Moreover, the adjusted item-total correlation was calculated [81,82,83]. In addition, item discriminant power (IDP) was performed to assess the capacity of the items to differentiate between individuals with a low or high level of the construct that has been measured [84,85]. For each subject, the total score and its quartile rank were first computed. Subsequently, independent sample *t*-tests—and related effect sizes (Cohen’s *d*) [86]—were determined to evaluate IDP by means of the scale-total score as the dependent variable and its lowest and highest quartile as the grouping variable [84,85].

Convergent validity was calculated with the Pearson correlation coefficient [81] and read using Cohen’s benchmarks: *r* < 0.10, trivial; *r* from 0.10 to 0.30, small; *r* from 0.30 to 0.50, moderate; and *r* > 0.50, large [86].

Moreover, a Receiver Operating Characteristics (ROC) curve was run to evaluate the accuracy of the general dimension of the AEBS-IT (‘addiction-like eating behavioral’ scale) to differentiate between (A) individuals without FA and individuals with FA as well as (B) individuals without BED and individuals with BED [87,88]. The global accuracy-validity of the AEBS-IT was estimated with the area under the ROC curve (AUC; 5000 stratified bootstrap resamples). The AUC was interpreted with Swets’ benchmarks: AUC = 0.50, null; AUC from 0.51 to 0.70, small; AUC from 0.71 to 0.90, moderate; AUC from 0.91 to 0.99, large; and AUC = 1.00, perfect accuracy [89,90]. Moreover, sensitivity (SE), specificity (SP), and accuracy (ACC) were computed for each AEBS-IT cut-off point [87,88].

## 3. Results

### 3.1. Structural Validity and Model Comparisons

The first model of the AEBS-IT (two first-order latent factors) showed a non-adequate fit to the data for the two samples combined. The Chi-square statistic was statistically significant: S-Bχ^2^ (89) = 2284.543; *p* < 0.001. The RMSEA was equal to 0.161; 90%CI: 0.155–0.167; *p*(RMSEA < 0.05) < 0.001. The CFI was equal to 0.945. The SRMR was equal to 0.109.

The second model of the AEBS-IT (one first-order latent factor) showed a non-adequate fit to the data for the two samples combined. The Chi-square statistic was statistically significant: S-Bχ^2^ (90) = 4505.043; *p* < 0.001. The RMSEA was equal to 0.227; 90%CI: 0.221–0.233; *p*(RMSEA < 0.05) < 0.001. The CFI was equal to 0.891. The SRMR was equal to 0.152.

**Figure 1:**
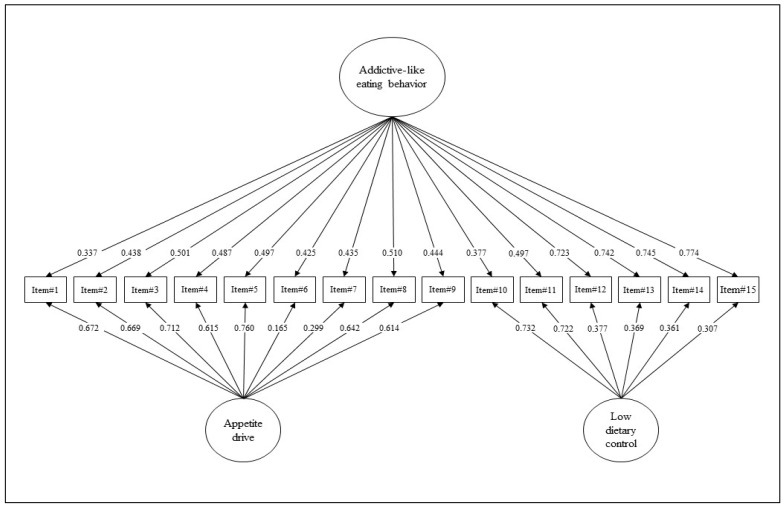
Structural model with factor loadings of the Italian AEBS—overall sample.

Lastly, the third model of the AEBS-IT (bi-factor model) showed an excellent fit with the data for the two samples combined. The Chi-square statistic was statistically significant: S-Bχ^2^ (75) = 506.245; *p* < 0.001. The RMSEA was equal to 0.078; 90%CI: 0.071–0.084; *p*(RMSEA < 0.05) < 0.001. The CFI was equal to 0.989. The SRMR was equal to 0.056. (Figure 1 and Table 1).

Model comparisons analysis showed the superiority of the bi-factor model (Table 2). Thus, the third model was considered the best factorial structure of the AEBS-IT. Consequently, the bi-factor model was used for subsequent statistical analyses.

### 3.2. Measurement Invariance across Samples

Inpatients with severe obesity. The Chi-square statistic was statistically significant: χ^2^ (75) = 233.036; *p* < 0.001. However, the RMSEA [RMSEA = 0.065; 90%CI: 0.056–0.074; *p*(RMSEA < 0.05) = 0.005], the CFI (CFI = 0.993), and the SRMR (SRMR = 0.050) were indicative of a good model fit.

General population. The Chi-square statistic was statistically significant: χ^2^ (75) = 282.602; *p* < 0.001. However, the RMSEA [RMSEA = 0.078; 90%CI: 0.069–0.088; *p*(RMSEA < 0.05) < 0.001], the CFI (CFI = 0.989), and the SRMR (SRMR = 0.065) were indicative of a good model fit.

Configural Invariance. The configural invariance model showed good model fit indices: χ^2^ (150) = 515.638, *p* < 0.001; the RMSEA = 0.072; the CFI = 0.991; and the SRMR = 0.057; signifying a similar factor structure between inpatients with severe obesity and the community sample.

Metric Invariance. The metric invariance model fitted the data well: χ^2^ (180) = 847.357, *p* < 0.001; the RMSEA = 0.088; the CFI = 0.984; and the SRMR = 0.074. A statistically significant decrease in Chi-square was found: DIFTEST (30) = 331.72; *p* < 0.001. However, a *non*-statistically significant decrease in CFI (|ΔCFI| = 0.007) was found—indicating that items were equivalently related to the latent factor independent of the sample.

Scalar Invariance. The scalar invariance model showed almost-good model fit indices: χ^2^ (222) = 1282.820, *p* < 0.001; the RMSEA = 0.100; the CFI = 0.975; and the SRMR = 0.073. A statistically significant decrease in Chi-square was detected: DIFTEST (42) = 435.46; *p* < 0.001. However, a *non*-statistically significant decrease in CFI (|ΔCFI| = 0.009) was detected—suggesting that inpatients with severe obesity and individuals from the general population had the same expected item response at the same absolute level of the trait.

Latent Means Invariance. The latent mean invariance model fitted the data well: χ^2^ (225) = 1820.760, *p* < 0.001; the RMSEA = 0.122; the CFI = 0.962; and the SRMR = 0.073. The Chi-square showed a statistically significant decrease: DIFTEST (3) = 537.94; *p* < 0.001. Also, a statistically significant decreases in CFI (|ΔCFI| = 0.013) was detected—signifying a difference in the expected latent mean of the traits of the two samples.

### 3.3. Psychometrics Properties

The IDP analysis showed that 15 items comprising the AEBS-IT discriminated between subjects with low and high addiction-like eating behaviors well in both specific factors (‘appetite drive’ scale and ‘low dietary control’ scale) and the general dimension (Table 1). For the ‘appetite drive’ factor, the lower discriminative item was item#9 (*t*_i_ = −13.42, *p* < 0.001, *d* = 1.25), while the higher discriminative item was item#5 (*t*_i_ = −36.76, *p* < 0.001, *d* = 3.40). For the ‘low dietary control’ factor, the lower discriminative item was item#14 (*t*_i_ = −22.13, *p* < 0.001, *d* = 1.99) while the higher discriminative item was item#12 (*t*_i_ = −34.58, *p* < 0.001, *d* = 3.12). For the ‘addiction-like eating behavior’ general dimension, the lower discriminative item was item#9 (*t*_i_ = −13.05, *p* < 0.001, *d* = 1.17), while the higher discriminative item was item#5 (*t*_i_ = −28.69, *p* < 0.001, *d* = 2.57).

In addition, the adjusted item-total correlation revealed statistically significant associations between each item and their specific factor as well as the general dimension (Table 1).

Reliability analysis showed substantial outcomes: for the ‘appetite drive’ scale, Cronbach’s alpha and McDonald’s ω were equal to 0.880 and to 0.922, respectively; for the ‘low dietary control’ scale, Cronbach’s alpha resulted equal to 0.787, and McDonald’s ω resulted equal to 0.906; for the ‘addiction-like eating behavior’ scale (total score), Cronbach’s alpha corresponded to 0.883, and McDonald’s corresponded to 0.919. 

### 3.4. Convergent Validity

Moderate-to-large correlations were found between the AEBS-IT ‘appetite drive’ scale and the mYFAS2.0 symptom count (*r* = 0.637, *p* < 0.001), the BES total score scale (*r* = 0.780; *p* < 0.001), the BED FC scale (*r* = 0.705; *p* < 0.001), the BES B scale (*r* = 0.766, *p* < 0.001), the MEC10-IT (*r* = 0.754; *p* < 0.001), the DEBQ total score scale (*r* = 0.561; *p* < 0.001), the DEBQ EE scale (*r* = 0.643; *p* < 0.001), and the DEBQ ExE scale (*r* = 0.338; *p* < 0.001). Moreover, the correlation between the ‘appetite drive’ scale and the BMI was *r* = 0.204, *p* < 0.001.

Small-to-moderate correlations resulted between the ‘low dietary control’ scale and the mYFAS2.0 symptom count (*r* = 0.373, *p* < 0.001), the BES total score scale (*r* = 0.520; *p* < 0.001), the BED FC scale (*r* = 0.465; *p* < 0.001), the BES B scale (*r* = 0.516, *p* < 0.001), the MEC10-IT (*r* = 0.447; *p* < 0.001), the DEBQ total score scale (*r* = 0.182; *p* = 0.015), the DEBQ RE scale (*r* = -0.167; *p* = 0.025), and the DEBQ EE scale (*r* = 0.339; *p* < 0.001). Moreover, the correlation between the ‘low dietary control’ scale and the BMI was *r* = 0.189, *p* < 0.001.

**Figure 2:**
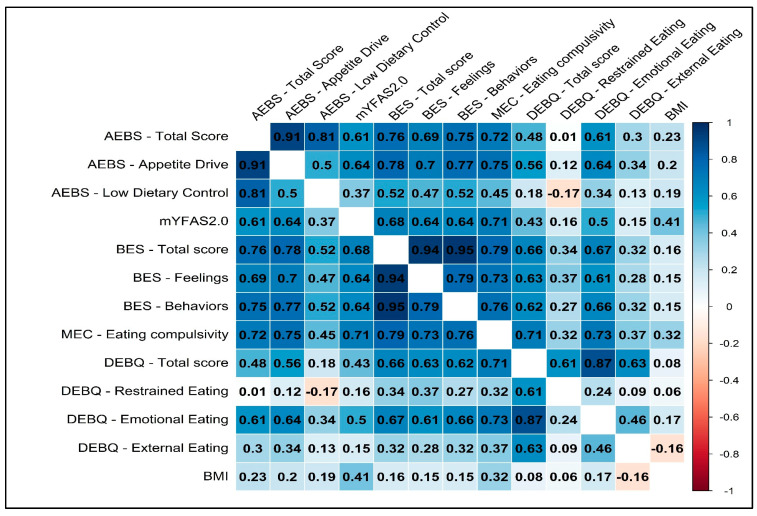
Correlations among variables.

Moderate-to-large correlations were found between the AEBS-IT ‘addiction-like eating behavior’ scale and the mYFAS2.0 symptom count (*r* = 0.606, *p* < 0.001), the BES total score scale (*r* = 0.765; *p* < 0.001), the BED FC scale (*r* = 0.689; *p* < 0.001), the BES B scale (*r* = 0.754, *p* < 0.001), the MEC10-IT (*r* = 0.719; *p* < 0.001), the DEBQ total score scale (*r* = 0.477; *p* < 0.001), the DEBQ EE scale (*r* = 0.608; *p* < 0.001), and the DEBQ ExE scale (*r* = 0.298; *p* < 0.001). Moreover, the correlation between the ‘addiction-like eating behavior’ scale and the BMI was *r* = 0.227, *p* < 0.001. Results are reported in Figure 2.

### 3.5. Accuracy of the ‘Addiction-Like Eating Behavior’ Scale as a Screening/Diagnostic Tool

**Figure 3:**
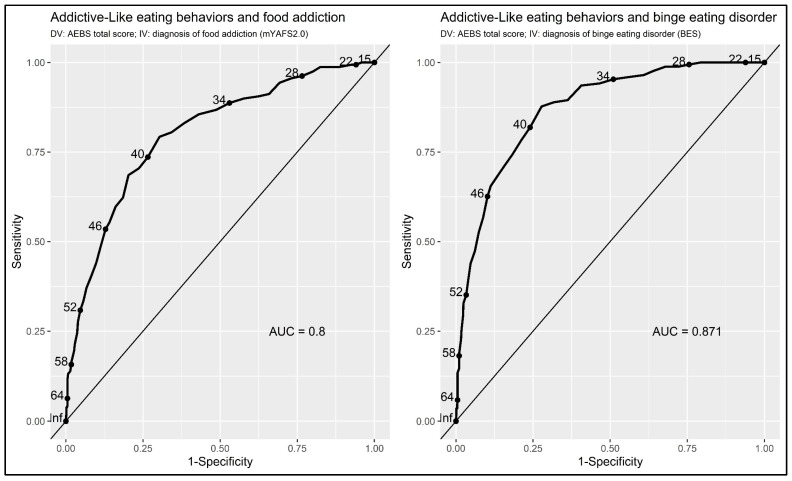
ROC curves.

The ‘addiction-like eating behavior’ scale (i.e., AEBS-IT total score) resulted highly accurate in distinguishing between individuals with/without FA: AUC = 0.819, se = 0.018; 95%CI = 0.783–0.855; *p* < 0.001 (Figure 3—left figure). With a cut-off point of 38 (i.e., AEBS-IT ≥ 39: risk of FA), ROC curves showed a SE = 0.807 (95%CI: 0.747–0.867), a SP = 0.701 (95%CI: 0.669–0.733), and an ACC = 0.720 (95%CI: 0.719–0.720). Based on the gold standard test (mYFAS2.0), 82.6% of individuals were classified as non-food-addicted and 17.4% of individuals were considered food-addicted (overall sample = 953). Thus, using the reported cut-off of 38 for the ‘addiction-like eating behavior’ scale, ROC curves showed that 57.9% of respondents were properly considered as ‘true negative’ and 14.1% as ‘true positive’ (72% of correct classifications). On the contrary, 3.4% were classified as ‘false negative’ and 24.7% were classified as ‘false positive’ (28% of misclassifications).

The ‘addiction-like eating behavior’ scale (i.e., AEBS-IT total score) reached excellent accurateness in discriminating between individuals with/without BED: AUC = 0.895, se = 0.013; 95%CI = 0.869–0.920; *p* < 0.001 (Figure 3— right figure). With a cut-off point of 38 (i.e., AEBS-IT ≥ 39: risk of BED), ROC curves showed a SE = 0.885 (95%CI: 0.840–0.930), a SP = 0.738 (95%CI: 0.706–0.769), and an ACC = 0.767 (95%CI: 0.767–0.767). Based on the gold standard test (BES), 71.7% of individuals were considered as non-binge eaters and 28.3% of individuals were considered as binge eaters (overall sample = 953). Thus, using the reported cut-off of 38 for the ‘addiction-like eating behavior’ scale, ROC curves showed that 53.3% of respondents were properly considered as ‘true negative’ and 25.2% as ‘true positive’ (78.5% of correct classifications). On the contrary, 3.1% were classified as ‘false negative’ and 18.4% were classified as ‘false positive’ (21.5% of misclassifications).

## 4. Discussion

The use of the AEBS-IT is important in both research and clinical fields as it is the only tool available enabling the evaluation of behavioral addiction to eating.

This study investigates, for the first time, the psychometric proprieties of this tool among the Italian population and tests its factorial structure, also in the comparison between a clinical sample of adults with obesity seeking treatment for weight reduction and the general population. 

In terms of the two samples combined, results confirmed that the bi-factor model of the AEBS-IT has an excellent fit to the data—meaning the nature of the construct of behavioral addiction to eating is adequately measured with the items comprising the tool.

MI analysis also revealed this to be true in both clinical and community samples, separately. The AEBS-IT’s items were equivalently related to the latent factor in each sample, and the two samples had the same expected item response at the same absolute level of the trait. These results suggest that inpatients with obesity and the general population interpreted the items in the same way (the factorial structure was equal across samples), with the same strength (items were related to the latent construct equally between the two samples), and with the same starting point (item thresholds were equal between the two samples). However, the latent trait was not equally distributed (latent means were different between the two samples). Thus, inpatients with severe obesity and the general population were perfectly comparable (equal items threshold), but with caution (different latent means) [91,92,93,94,95]. Therefore, the AEBS-IT can be employed in clinical and research practice to confront outcomes resulting from these two populations.

Moreover, IDP analysis exhibited that the 15 items of the AEBS-IT discriminated well between respondents with low and high addiction-like eating behaviors, and demonstrated the capacity of each item to signify its latent construct.

Further, reliability analyses were run, showing good results for both subscales and the AEBS-IT total score. Statistically significant positive correlations were also found between all the dimensions of the AEBS-IT, the mYFAS2.0 symptom count, the BES subscales, the MEC10-IT total score, the DEBQ factors, and the individuals’ BMI—demonstrating the tool’s good convergent validity.

These findings corroborate the link between the construct of addiction-like eating behaviors, FA, BED symptoms, compulsive eating, dysfunctional eating patterns, and the individuals’ BMI.

Not surprisingly, the highest correlations were observed between the AEBS-IT scores, and both the mYFAS2.0 symptom count and BES scores—thus confirming the finding of the validation study carried out by Legendre et al. (2020) in both clinical and community samples among Canadian adults [26].

Further, in a recent examination of the YFAS in a clinical sample of patients with obesity with BED, a diagnosis of FA was met by 57% of patients, and a higher number of FA symptoms was related to more frequent binge-eating episodes [96,97].

Additionally, the compulsive overeating characteristic of BED has been shown to statistically predict FA diagnosis based on YFAS criteria in individuals with obesity in several investigations [98,99].

These results corroborate the partial overlap between the constructs of compulsive eating measured using the MEC10-IT, with a diagnosis of FA based on addiction criteria and BED already documented in the previous studies [26,49,100,101], and also highlight the important relationships between addiction-like eating behaviors with BED and compulsive eating patterns. Indeed, the AEBS-IT total score and its subscales showed higher correlations with all the BES dimensions and the construct of eating compulsivity measured using the MEC10-IT than those displayed by the mYFAS2.0 symptom count, while FA measured using the mYFAS2.0 and FA based on the behavioral models show a moderate correlation. The lowest correlations were, instead, observed between the AEBS-IT subscales and total score and the DEBQ restrained eating dimension. Specifically, a negative correlation was detected between the AEBS-IT low dietary control factor and the above DEBQ dimension. Accordingly, previous investigations showed small, significant negative correlations or non-significant correlation coefficients of FA based on the substance abuse model (measured with the YFAS) with dietary restraint (measured with the Three-Factor Eating Questionnaire—TFEQ or the Eating Disorder Examination—EDE) [12,96,102].

Still, for the first time, the construct of EA is measured here through the AEBS-IT, while the presence of eating disorder psychopathology was assessed using the DEBQ.

These findings further corroborate the association with mYFAS2.0 and AEBS-IT conceptualization of EA. Still, whether these conditions represent forms of compulsive eating supported by different mechanisms or represent two different facets of a unique underlying phenomenon needs to be further assessed in future studies. In fact, the heterogeneity characterizing the sample (i.e., diagnosis and demographics/clinical parameters) and the cross-sectional design of this study make it difficult to derive any conclusion or causal relation between variables.

Lastly, the ROC analyses revealed that the AEBS-IT represents a valid screening/diagnostic tool for the detection of addiction-like eating behaviors in people with severe obesity. Indeed, it presented high accuracy (AUC = 0.819), sensitivity (0.807), and specificity (0.701) in discriminating between individuals with FA and those without FA. Similarly, the measure demonstrated to be able to successfully detect BED symptoms among adults with severe obesity seeking treatment for weight reductions—as it showed high accuracy (AUC = 0.895), sensitivity (0.885), and high specificity (0.738) in discriminating among individuals with and without BED.

In this regard, the magnitude of the effect size (AUC) suggests a substantial (but not total) overlap between food addiction, binge-eating behaviors, and eating addiction, suggesting that these three psychological constructs may be somewhat intersecting and not necessarily mutually exclusive. Indeed, food addiction—conceptualized as SUD—does not necessarily exclude overeating and binge-eating behaviors and—at the same time—does not necessarily exclude a drive toward hunger (appetitive drive) that can lead to low diet control through behavioral dependence on the act of eating.

Some limitations of this study should be highlighted. Despite the presence of a large number of subjects from the clinical population (inpatients with severe obesity), a convenience snowball sampling enrollment procedure was used for the individuals from the general population. However, MI analysis suggests that the factorial structure of AEBS is invariant across the two samples, at the level of the thresholds. Moreover, the cross-sectional research design and the use of self-report questionnaires did not allow for testing of the possible changes of the AEBS over time nor its temporal stability (e.g., longitudinal MI and test-retest reliability) and its predictive validity. Future studies may identify the recurring patterns of EA by creating latent psychological profiles. Another potential limitation is related to the variability in the age of both samples—which could lead to changes in the subjects’ metabolism and, thus, their tendency to engage in EA-related behaviors. Future studies, including longitudinal ones, could control for this variable and use it as a predictor/outcome to create possible explanatory models of EA behaviors. In addition, future studies could investigate the interaction of confounding factors (e.g., age, BMI, gender, etc.) on the latent dimensions of AEBS. Lastly, future studies might also consider examining possible cross-cultural similarities and differences in the conceptualization of EA through AEBS (i.e., cross-cultural MI).

Despite these limitations, this study still has several strengths, both methodological and clinical. It is noteworthy that this is the first study validating the AEBS-IT, allowing for the assessment of EA to be conducted with accuracy and parsimony. Additionally, the relatively small number of items allows the AEBS-IT to be more easily included in longer assessment batteries, both in clinical and research practice. About the methodological strengths, the CFA revealed that the best factorial of the AEBS-IT is the one comprising two specific factors (‘appetite drive’ and ‘low dietary control’) and an overarching latent dimension (‘eating addiction’). Regarding clinical strengths, the AEBS-IT has significant clinical impact and implications, as it represents a useful assessment tool for clinicians because EA seems to be a transdiagnostic construct shared by various EDs and psychological difficulties related to eating and feeding attitudes. Thus, assessing EA is important due to its multiple roles: EA may be the cause, the result, and a maintenance factor of psychological suffering and dysfunctional behaviors in EDs, and this provides useful information for both the conceptualization and treatment of clinical conditions. Moreover, the AEBS-IT provides useful information to allow for better understandings of psychological difficulties and the tailoring of specific psychological interventions.

## 5. Conclusions

Conceptualizing FA as a behavioral addiction—namely, ‘eating addiction’ (EA)—the AEBS-IT represents a psychometrically sound instrument able to measure the presence of addictive-like eating behavioral patterns in both clinical and nonclinical samples. Indeed, this tool demonstrated good validity and reliability in both patients with severe obesity and the community sample, and might be used by researchers and clinicians to assess FA [103]. Considering that the AEBS-IT—unlike the YFAS/YFAS2.0/mYFAS2.0—is not meant to be a diagnostic tool, its good sensitivity to clinical populations (i.e., a good capacity to detect people with FA) further supports its utilization. Additionally—besides being moderately correlated with the FA-substance-based model—it shows high associations with compulsive eating, BED, and dysfunctional eating patterns that are above those displayed by the mYFAS 2.0. This suggests that, despite the YFAS representing the most widely used measure of FA, the AEBS-IT nonetheless properly reflects the behavioral correlates of the EA phenomenon (i.e., compulsive overeating), its characteristics, and related psychiatric comorbidities. Moreover, the term “Eating Addiction” would be more appropriate for describing the behavioral phenomenon of continuous overeating of a variety of foods and avoiding the conflicting assumptions that certain food can lead to the development of a SUD.

Still, the scientific debate about “eating addiction” is in its infancy, and further studies should try to replicate these results by also employing cross-cultural designs and investigating the AEBS discrimination capability with a wider range of populations, including those with BED and other eating disorders.

## Figures and Tables

**Table 1:** Descriptive statistics, psychometric properties of each item, and results of the confirmatory factor analysis (CFA).

Overall Sample (*N* = 953)													
	Descriptive Statistics				IDP—Specific Factor			IDP—General Factor			CFA		
	Mean	SD	SK	K	*t_sf_*	*d_sf_*	*r*_(it−tot) *sf*_	*t_gf_*	*d_gf_*	*r*_(it−tot) *gf*_	|λ*_sf_|*	|λ*_gf_*|	*R* ^2^
Item#1	1.905	0.995	1.250	1.227	−20.79	1.93	0.640	−17.58	1.57	0.546	0.672	0.337	0.565
Item#2	2.065	1.073	1.021	0.401	−26.87	2.49	0.692	−22.03	1.97	0.624	0.669	0.438	0.639
Item#3	2.114	1.100	0.963	0.194	−32.64	3.02	0.781	−26.58	2.38	0.704	0.712	0.501	0.758
Item#4	2.142	1.255	0.906	−0.336	−33.41	3.10	0.710	−26.11	2.34	0.646	0.615	0.487	0.615
Item#5	2.151	1.100	0.875	−0.023	−36.76	3.40	0.793	−28.69	2.57	0.713	0.760	0.497	0.824
Item#6 *	3.329	1.261	−0.368	−0.991	−15.89	1.43	0.304	−16.35	1.46	0.401	0.165	0.425	0.208
Item#7	2.610	1.340	0.389	−1.084	−19.30	1.77	0.414	−17.77	1.59	0.440	0.299	0.435	0.279
Item#8	1.751	1.052	1.561	1.824	−24.38	2.27	0.729	−21.85	1.96	0.654	0.642	0.510	0.672
Item#9	1.363	0.797	2.714	7.653	−13.42	1.25	0.565	−13.05	1.17	0.499	0.614	0.444	0.574
Item#10 *	2.768	1.245	0.295	−0.892	−25.40	2.28	0.483	−14.28	1.28	0.323	0.732	0.377	0.678
Item#11 *	2.898	1.182	0.133	−0.870	−30.45	2.74	0.581	−20.05	1.79	0.450	0.722	0.497	0.768
Item#12 *	2.919	1.223	0.203	−0.920	−34.58	3.12	0.637	−24.10	2.15	0.554	0.377	0.723	0.665
Item#13 *	2.803	1.151	0.299	−0.710	−31.09	2.78	0.642	−23.45	2.10	0.582	0.369	0.742	0.687
Item#14	2.910	1.155	−0.065	−0.933	−22.13	1.99	0.418	−18.19	1.63	0.474	0.361	0.745	0.686
Item#15	2.965	1.220	−0.128	−1.049	−24.42	2.21	0.466	−21.47	1.92	0.513	0.307	0.774	0.693
**Inpatients with SEVERE obesity (*n* = 502)**													
	**Descriptive Statistics**				**IDP—Specific Factor**			**IDP—General Factor**			**CFA**		
	**Mean**	**SD**	**SK**	**K**	***t_sf_***	***d_sf_***	***r*_(it−tot) *sf*_**	***t_gf_***	***d_gf_***	***r*_(it−tot) *gf*_**	**|λ*_sf_|***	**|λ*_gf_*|**	***R* ^2^**
Item#1	1.793	1.009	1.441	1.638	−15.65	1.98	0.668	−14.44	1.85	0.604	0.595	0.492	0.596
Item#2	2.203	1.149	0.881	−0.069	−21.60	2.72	0.710	−18.76	2.41	0.648	0.660	0.484	0.670
Item#3	2.203	1.108	0.829	−0.059	−24.36	3.06	0.780	−20.34	2.61	0.719	0.705	0.528	0.775
Item#4	2.380	1.333	0.603	−0.919	−29.77	3.74	0.734	−24.07	3.09	0.675	0.635	0.519	0.673
Item#5	2.231	1.163	0.743	−0.436	−31.37	3.93	0.796	−23.78	3.05	0.727	0.722	0.552	0.826
Item#6 *	3.424	1.286	−0.535	−0.847	−8.83	1.06	0.301	−10.69	1.38	0.405	0.104	0.448	0.211
Item#7	3.084	1.288	−0.027	−1.180	−16.08	1.96	0.428	−11.63	1.50	0.451	0.328	0.426	0.289
Item#8	1.922	1.156	1.248	0.642	−20.90	2.65	0.734	−19.17	2.46	0.669	0.585	0.565	0.662
Item#9	1.408	0.868	2.565	6.468	−10.50	1.34	0.559	−10.25	1.31	0.499	0.562	0.496	0.562
Item#10*	2.743	1.309	0.280	−1.029	−18.48	2.32	0.462	−12.05	1.55	0.356	0.639	0.448	0.609
Item#11 *	2.875	1.228	0.039	−0.925	−21.96	2.78	0.591	−16.71	2.15	0.482	0.668	0.557	0.756
Item#12 *	3.223	1.303	−0.191	−1.023	−23.19	2.94	0.587	−16.76	2.16	0.514	0.424	0.659	0.614
Item#13 *	2.994	1.228	0.076	−0.915	−24.67	3.11	0.598	−17.22	2.22	0.549	0.410	0.681	0.632
Item#14	3.129	1.204	−0.333	−0.878	−12.17	1.55	0.340	−11.43	1.47	0.418	0.454	0.656	0.636
Item#15	3.124	1.272	−0.321	−0.975	−13.28	1.70	0.391	−14.18	1.83	0.495	0.495	0.777	0.848
**General Population (*n* = 451)**													
	**Descriptive Statistics**				**IDP—Specific Factor**			**IDP—General Factor**			**CFA**		
	**Mean**	**SD**	**SK**	**K**	***t_sf_***	***d_sf_***	***r*_(it−tot) *sf*_**	***t_gf_***	***d_gf_***	***r*_(it−tot) *gf*_**	**|λ*_sf_|***	**|λ*_gf_*|**	***R* ^2^**
Item#1	2.029	0.965	1.107	1.054	−16.11	2.18	0.718	−12.97	1.73	0.582	0.770	0.299	0.683
Item#2	1.911	0.960	1.133	0.977	−17.18	2.33	0.648	−14.01	1.87	0.562	0.681	0.348	0.584
Item#3	2.016	1.084	1.136	0.612	−21.97	2.97	0.788	−17.04	2.28	0.691	0.717	0.482	0.746
Item#4	1.876	1.105	1.311	0.935	−16.00	2.17	0.648	−13.32	1.78	0.562	0.620	0.384	0.531
Item#5	2.062	1.020	1.023	0.604	−23.64	3.20	0.797	−18.10	2.42	0.698	0.783	0.444	0.810
Item#6 *	3.224	1.226	−0.187	−1.074	−12.76	1.72	0.289	−12.92	1.72	0.383	0.203	0.391	0.194
Item#7	2.082	1.193	1.002	0.075	−10.11	1.37	0.362	−9.02	1.20	0.360	0.325	0.329	0.213
Item#8	1.561	0.886	2.002	4.243	−12.60	1.71	0.703	−11.32	1.51	0.597	0.720	0.407	0.683
Item#9	1.313	0.707	2.844	9.003	−8.40	1.14	0.576	−7.99	1.07	0.497	0.662	0.392	0.592
Item#10 *	2.796	1.171	0.336	−0.714	−18.09	2.40	0.547	−9.74	1.30	0.306	0.904	0.271	0.891
Item#11 *	2.925	1.129	0.279	−0.842	−20.65	2.73	0.610	−12.93	1.72	0.443	0.738	0.420	0.721
Item#12 *	2.581	1.026	0.562	−0.209	−20.44	2.70	0.698	−15.25	2.04	0.564	0.386	0.758	0.724
Item#13 *	2.590	1.019	0.483	−0.307	−20.55	2.71	0.683	−15.66	2.09	0.595	0.356	0.796	0.760
Item#14	2.665	1.046	0.153	−0.742	−16.29	2.14	0.487	−12.43	1.66	0.505	0.118	0.787	0.634
Item#15	2.789	1.136	0.046	−1.038	−19.94	2.64	0.541	−15.45	2.06	0.508	0.000	0.744	0.554

Notes: * = item reverse (reversed); SD = standard deviation; SK = skewness; K = kurtosis; (…)*sf* = referred to the specific factor (item#1 to item#9: appetite drive; item#10 to item#15: low dietary control); (…)*gf* = referred to the general factor; IDP = item discriminant power; *t* = *t*-test; *d* = Cohen’s d; *r*_(IT-TOT)_ = item-total correlation (adjusted); |λ*sf*| = absolute value of the factor loading on the specific factor (item#1 to item#9: appetite drive; item#10 to item#15: low dietary control); |λ*gf*| = absolute value of the factor loading on the general factor; *R*^2^ = explained variance.

**Table 2:** Model comparisons.

Model	χ^2^ (*df*)	CFI	RMSEA	Comparison	Δχ^2^(Δ*df*)	*p*-Value	|ΔCFI|	|ΔRMSEA|
1. Bi-factor model	506.245 (75)	0.989	0.078					
2. Two first-order factors model	2284.543 (89)	0.945	0.161	1 vs. 2	1178.3 (14)	< 0.001	0.045	0.083
3. Single factor model	4505.043 (90)	0.891	0.227	1 vs. 3	3998.8 (15)	< 0.001	0.099	0.149

Note. χ^2^ = Satorra-Bentler scaled Chi-square test; *df* = degree of freedoms; Δ = differences between indices; RMSEA = root mean square error of approximation; CFI = comparative fit index.

## Data Availability

The data presented in this study are available on request from the corresponding author. The data are not publicly available due to privacy restrictions.

## Appendix A

                                                        AEBS

**ISTRUZIONI:** Questo questionario contiene una serie di affermazioni relative ad **atteggiamenti**, **sentimenti** o **comportamenti legati al cibo**, di cui le persone possono fare esperienza. Segna la risposta che meglio ti descrive **NON ESISTONO RISPOSTE GIUSTE O SBAGLIATE**.

Part I:

1	2	3	4	5
MAI/QUASI MAI	QUALCHE VOLTA	METÀ DELLE VOLTE	IL PIÙ DELLE VOLTE	QUASI SEMPRE/ SEMPRE

1	Continuo a mangiare nonostante mi senta pieno	1	2	3	4	5
2	Mi servo porzioni eccessivamente grandi	1	2	3	4	5
3	Trovo difficile limitare cosa e/o quanto mangio	1	2	3	4	5
4	Una volta che comincio a mangiare certi cibi, non posso fermarmi finché non rimane niente	1	2	3	4	5
5	Quando si tratta di cibo, tendo a esagerare	1	2	3	4	5
6	Tendo a *non* mangiare troppo	1	2	3	4	5
7	Mi sento incapace di controllare il mio peso	1	2	3	4	5
8	Faccio abbuffate (grandi quantità di cibo in poco tempo)	1	2	3	4	5
9	Mangio fino a quando mi sento male	1	2	3	4	5

Part II:

1	2	3	4	5
FORTEMENTE IN DISACCORDO	IN DISACCORDO	NÉ IN ACCORDO, NÉ IN DISACCORDO	IN ACCORDO	FORTEMENTE IN ACCORDO

10	Tendo a *non* comprare alimenti lavorati ad alto contenuto di grassi e/o zuccheri	1	2	3	4	5
11	*Non* mangio molti cibi ad alto contenuto di grassi e/o zuccheri	1	2	3	4	5
12	Credo di avere una dieta sana	1	2	3	4	5
13	Riesco facilmente a fare scelte alimentari sane	1	2	3	4	5
14	Nonostante provi a mangiare in modo sano, finisco per mangiare cibi “cattivi”	1	2	3	4	5
15	Nonostante sia consapevole dell’effetto sulla mia salute, continuo a mangiare certi cibi non salutari	1	2	3	4	5

## References

[1] World Health Organisation Obesity and Overweight. https://www.who.int/news-room/fact-sheets/detail/obesity-and-overweight.

[2] Avila C., Holloway A.C., Hahn M.K., Morrison K.M., Restivo M., Anglin R., Taylor V.H. (2015). An overview of links between obesity and mental health. Curr. Obes. Rep..

[3] Rajan T.M., Menon V. (2017). Psychiatric disorders and obesity: A review of association studies. J. Postgrad. Med..

[4] Campbell M.K. (2016). Biological, environmental, and social influences on childhood obesity. Pediatr. Res..

[5] Pietrabissa G. (2018). Group Motivation-Focused Interventions for Patients with Obesity and Binge Eating Disorder. Front. Psychol..

[6] De Lorenzo A., Romano L., Di Renzo L., Di Lorenzo N., Cenname G., Gualtieri P. (2020). Obesity: A preventable, treatable, but relapsing disease. Nutrition.

[7] Hebebrand J., Albayrak O., Adan R., Antel J., Dieguez C., de Jong J., Leng G., Menzies J., Mercer J.G., Murphy M. (2014). “Eating addiction”, rather than “food addiction”, better captures addictive-like eating behavior. Neurosci. Biobehav. Rev..

[8] Meule A., Heckel D., Jurowich C.F., Vogele C., Kubler A. (2014). Correlates of food addiction in obese individuals seeking bariatric surgery. Clin. Obes..

[9] Gearhardt A.N., Corbin W.R., Brownell K.D. (2009). Preliminary validation of the Yale Food Addiction Scale. Appetite.

[10] Manzoni G.M., Rossi A., Pietrabissa G., Varallo G., Molinari E., Poggiogalle E., Donini L.M., Tarrini G., Melchionda N., Piccione C. (2018). Validation of the Italian Yale Food Addiction Scale in postgraduate university students. Eat. Weight Disord..

[11] American Psychiatric Association (2000). Diagnostic and Statistical Manual of Mental Disorders.

[12] Pursey K.M., Gearhardt A.N., Burrows T.L. (2016). The relationship between "food addiction" and visceral adiposity in young females. Physiol. Behav..

[13] Meule A., Gearhardt A.N. (2019). Ten Years of the Yale Food Addiction Scale: A Review of Version 2.0. Curr. Addict. Rep..

[14] Aloi M., Rania M., Rodriguez Munoz R.C., Jimenez Murcia S., Fernandez-Aranda F., De Fazio P., Segura-Garcia C. (2017). Validation of the Italian version of the Yale Food Addiction Scale 2.0 (I-YFAS 2.0) in a sample of undergraduate students. Eat. Weight. Disord..

[15] Manzoni G.M., Rossi A., Pietrabissa G., Mannarini S., Fabbricatore M., Imperatori C., Innamorati M., Gearhardt A.N., Castelnuovo G. (2021). Structural validity, measurement invariance, reliability and diagnostic accuracy of the Italian version of the Yale Food Addiction Scale 2.0 in patients with severe obesity and the general population. Eat. Weight. Disord. Stud. Anorex. Bulim. Obes..

[16] American Psychiatric Association (2013). Diagnostic and Statistical Manual of Mental Disorders.

[17] Burrows T., Kay-Lambkin F., Pursey K., Skinner J., Dayas C. (2018). Food addiction and associations with mental health symptoms: A systematic review with meta-analysis. J. Hum. Nutr. Diet.

[18] Ivezaj V., Wiedemann A.A., Grilo C.M. (2017). Food addiction and bariatric surgery: A systematic review of the literature. Obes. Rev..

[19] Pursey K.M., Stanwell P., Gearhardt A.N., Collins C.E., Burrows T.L. (2014). The Prevalence of Food Addiction as Assessed by the Yale Food Addiction Scale: A Systematic Review. Nutrients.

[20] Ziauddeen H., Fletcher P.C. (2013). Is food addiction a valid and useful concept?. Obes. Rev. Off. J. Int. Assoc. Study Obes..

[21] Ruddock H.K., Christiansen P., Halford J.C.G., Hardman C.A. (2017). The development and validation of the Addiction-like Eating Behaviour Scale. Int. J. Obes..

[22] Markus C.R., Rogers P.J., Brouns F., Schepers R. (2017). Eating dependence and weight gain; no human evidence for a ’sugar-addiction’ model of overweight. Appetite.

[23] Ziauddeen H., Farooqi I.S., Fletcher P.C. (2012). Obesity and the brain: How convincing is the addiction model?. Nat. Rev. Neurosci..

[24] Bechara A. (2005). Decision making, impulse control and loss of willpower to resist drugs: A neurocognitive perspective. Nat. Neurosci..

[25] Wiers R.W., Bartholow B.D., van den Wildenberg E., Thush C., Engels R.C., Sher K.J., Grenard J., Ames S.L., Stacy A.W. (2007). Automatic and controlled processes and the development of addictive behaviors in adolescents: A review and a model. Pharmacol. Biochem. Behav..

[26] Legendre M., Begin C. (2021). French validation of the addiction-like eating behavior scale and its clinical implication. Eat. Weight Disord..

[27] Cardoso T.Q., Pereira C.W., de Souza Costa T., da CostaLima M.D. (2020). Translation and validation of the addiction-like Eating Behavior Scale from English to Portuguese in Brazil. J. Addict. Dis..

[28] Beaton D.E., Bombardier C., Guillemin F., Ferraz M.B. (2000). Guidelines for the process of cross-cultural adaptation of self-report measures. Spine.

[29] Guillemin F., Bombardier C., Beaton D.E. (1993). Cross-cultural adaptation of health-related quality of life measures: Literature review and proposed guidelines. J. Clin. Epidemiol..

[30] Hu L.T., Bentler P.M. (1999). Cutoff criteria for fit indexes in covariance structure analysis: Conventional criteria versus new alternatives. Struct. Equ. Model..

[31] Muthén B., Asparouhov T. (2002). Latent variable analysis with categorical outcomes: Multiple-group and growth modeling in Mplus. Mplus Web Notes.

[32] Yu C.Y. (2002). Evaluating Cutoff Criteria of Model Fit Indices for Latent Variable Models with Binary and Continuous Outcomes. Ph.D. Thesis.

[33] Tomarken A.J., Waller N.G. (2005). Structural Equation Modeling: Strengths, Limitations, and Misconceptions. Annu. Rev. Clin. Psychol..

[34] Flora D.B., Curran P.J. (2004). An empirical evaluation of alternative methods of estimation for confirmatory factor analysis with ordinal data. Psychol. Methods.

[35] Bentler P.M., Chou C.H. (1987). Practical issues in structural modeling. Soc. Methods Res..

[36] Boomsma A., Hoogland J.J., Cudeck R., du Toit S., Sorbom D. (2001). The robustness of LISREL modeling revisited. A Festschrift in honor of Karl Jöreskog. Structural Equation Models: Present and Future.

[37] Marsh H.W., Balla J.R., McDonald R.P. (1988). Goodness-of-fit indexes in confirmatory factor analysis: The effect of sample size. Psychol. Bull..

[38] Consoli S., Rossi A., Thompson L.Y., Volpi C., Mannarini S., Castelnuovo G., Molinari E. (2020). Assessing Psychometric Properties of the Italian Version of the Heartland Forgiveness Scale. Front. Psychol..

[39] Imperatori C., Fabbricatore M., Lester D., Manzoni G.M., Castelnuovo G., Raimondi G., Innamorati M. (2019). Psychometric properties of the modified Yale Food Addiction Scale Version 2.0 in an Italian non-clinical sample. Eat. Weight. Disord..

[40] Schulte E.M., Gearhardt A.N. (2017). Development of the Modified Yale Food Addiction Scale Version 2.0. Eur. Eat. Disord. Rev. J. Eat. Disord. Assoc..

[41] Gormally J., Black S., Daston S., Rardin D. (1982). The assessment of binge eating severity among obese persons. Addict. Behav..

[42] Ricca V., Mannucci E., Moretti S., Di Bernardo M., Zucchi T., Cabras P.L., Rotella C.M. (2000). Screening for binge eating disorder in obese outpatients. Compr. Psychiatry.

[43] Imperatori C., Innamorati M., Lamis D.A., Contardi A., Continisio M., Castelnuovo G., Manzoni G.M., Fabbricatore M. (2016). Factor Structure of the Binge Eating Scale in a Large Sample of Obese and Overweight Patients Attending Low Energy Diet Therapy. Eur. Eat. Disord. Rev. J. Eat. Disord. Assoc..

[44] Marcus M.D., Wing R.R., Hopkins J. (1988). Obese binge eaters: Affect, cognitions, and response to behavioural weight control. J. Consult. Clin. Psychol..

[45] Freitas S.R., Lopes C.S., Appolinario J.C., Coutinho W. (2006). The assessment of binge eating disorder in obese women: A comparison of the binge eating scale with the structured clinical interview for the DSM-IV. Eat. Behav..

[46] Grupski A.E., Hood M.M., Hall B.J., Azarbad L., Fitzpatrick S.L., Corsica J.A. (2013). Examining the Binge Eating Scale in screening for binge eating disorder in bariatric surgery candidates. Obes. Surg..

[47] Duarte C., Pinto-Gouveia J., Ferreira C. (2015). Expanding binge eating assessment: Validity and screening value of the Binge Eating Scale in women from the general population. Eat. Behav..

[48] Hood M.M., Grupski A.E., Hall B.J., Ivan I., Corsica J. (2013). Factor structure and predictive utility of the Binge Eating Scale in bariatric surgery candidates. Surg. Obes. Relat. Dis..

[49] Schroder R., Sellman J.D., Adamson S. (2017). Development and Validation of a Brief Measure of Eating Compulsivity (MEC). Subst. Use Misuse.

[50] Rossi A.A., Mannarini S., Schroder R., Castelnuovo G., Pietrabissa G. (2022). Eating Compulsivity in Inpatients with Severe Obesity and the General Population: The Italian Measure of Eating Compulsivity (MEC10-IT). Appetite.

[51] Van Strien T., Frijters J.E.R., Bergers G.P.A., Defares P.B. (1986). The Dutch Eating Behaviour Questionnaire (DEBQ) for assessment of restrained, emotional and external eating behaviour. Int. J. Eat. Disord..

[52] Dakanalis A., Zanetti M.A., Clerici M., Madeddu F., Riva G., Caccialanza R. (2013). Italian version of the Dutch Eating Behavior Questionnaire. Psychometric proprieties and measurement invariance across sex, BMI-status and age. Appetite.

[53] Van Strien T., Herman C.P., Verheijden M.W. (2013). Eating style, overeating and weight gain. A prospective 2-year follow-up study in a representative Dutch sample. Appetite.

[54] Riva G., Molinari E., Boringhieri B. (2004). Clinical Psychology of Obesity.

[55] R Core Team (2017). R: A Language and Environment for Statistical Computing.

[56] R Core Team (2014). The R Project for Statistical Computing.

[57] Wei T., Simko V. (2017). R Package “Corrplot”: Visualization of a Correlation Matrix, 0.84.

[58] Rosseel Y. (2012). lavaan: An R Package for Structural Equation Modeling. J. Stat. Softw..

[59] Rosseel Y., Oberski D., Byrnes J., Vanbrabant L., Savalei V., Merkle E., Hallquist M., Rhemtulla M., Katsikatsou M., Barendse M. (2015). Package ‘lavaan’.

[60] Sachs M.C. (2017). plotROC: A Tool for Plotting ROC Curves. J. Stat. Softw..

[61] Robin X., Turck N., Hainard A., Tiberti N., Lisacek F., Sanchez J., Müller M. (2011). pROC: An open-source package for R and S+ to analyze and compare ROC curves. BMC Bioinform..

[62] Revelle W. (2018). psych: Procedures for Personality and Psychological Research.

[63] Revelle W. (2020). psychTools: Tools to Accompany the ’Psych’ Package for Psychological Research, 2.0.6.

[64] semTools Contributors (2016). semTools: Useful Tools for Structural Equation Modeling, R package version 0.4-14.

[65] Wickham H., Averick M., Bryan J., Chang W., McGowan L.D., François R., Grolemund G., Hayes A., Henry L., Hester J. (2019). Welcome to the tidyverse. J. Open Source Softw..

[66] Wickham H. (2016). ggplot2: Elegant Graphics for Data Analysis.

[67] Brown T.A. (2015). Confirmatory Factor Analysis for Applied Research.

[68] Hoyle R.H. (2012). Handbook of Strucural Equation Modeling.

[69] Kline R.B. (2016). Principles and Practice of Structural Equation Modeling.

[70] Lionetti F., Keijsers L., Dellagiulia A., Pastore M. (2016). Evidence of factorial validity of parental knowledge, control and solicitation, and adolescent disclosure scales: When the ordered nature of Likert scales matters. Front. Psychol..

[71] Muthén L.K., Muthén B.O. (2017). Mplus User’s Guide.

[72] Van de Schoot R., Lugtig P., Hox J. (2012). A checklist for testing measurement invariance. Eur. J. Dev. Psychol..

[73] Cheung G.W., Rensvold R.B. (2002). Evaluating goodness-of-fit indexes for testing measurement invariance. Struct. Equ. Model..

[74] Millsap R.E. (2012). Statistical Approaches to Measurement Invariance.

[75] Millsap R.E., Yun-Tein J. (2004). Assessing Factorial Invariance in Ordered-Categorical Measures. Multivar. Behav. Res..

[76] Vandenberg R.J., Lance C.E. (2000). A Review and Synthesis of the Measurement Invariance Literature: Suggestions, Practices, and Recommendations for Organizational Research. Organ. Res. Methods.

[77] Meredith W. (1993). Measurement invariance, factor analysis and factorial invariance. Psychometrika.

[78] McDonald R.P. (1999). Test Theory: A Unified Treatment.

[79] McDonald R.P., Ho M.-H.R. (2002). Principles and practice in reporting structural equation analyses. Psychol. Methods.

[80] McDonald R.P., Mulaik S.A. (1979). Determinacy of common factors. Psychol. Bull..

[81] Tabachnick B.G., Fidell L.S. (2014). Using Multivariate Statistics.

[82] Pallant J. (2013). SPSS Survival Manual.

[83] Howell D.C. (2013). Statistical Methods for Psychology.

[84] Chiorri C. (2011). Teoria e Tecnica Psicometrica. Costruire un Test Psicologico.

[85] Ebel R.L. (1965). Measuring Educational Achievement.

[86] Cohen J. (1988). Statistical Power Analysis for the Behavioral Sciences.

[87] Pepe M.S. (2003). The Statistical Evaluation of Medical Tests for Classification and Prediction.

[88] Zhou X.H., Obuchowski N.A., McClish D. (2002). Statistical Methods in Diagnostic Medicine.

[89] Swets J.A. (1998). Measuring the accuracy of diagnostic systems. Science.

[90] Zweig M.H., Campbell G. (1993). Receiver-operating characteristic (ROC) plots: A fundamental evaluation tool in clinical medicine. Clin. Chem..

[91] Steiger J.H. (1990). Structural Model Evaluation and Modification: An Interval Estimation Approach. Multivar. Behav. Res..

[92] Bentler P.M. (1990). Comparative fit indexes in structural models. Psychol. Bull..

[93] Bentler P.M., Bonett D.G. (1980). Significance tests and goodness of fit in the analysis of covariance structures. Psychol. Bull..

[94] Manzoni G.M., Rossi A., Marazzi N., Agosti F., De Col A., Pietrabissa G., Castelnuovo G., Molinari E., Sartorio A. (2018). Feasibility, Validity, and Reliability of the Italian Pediatric Quality of Life Inventory Multidimensional Fatigue Scale for Adults in Inpatients with Severe Obesity. Obes. Facts.

[95] Pietrabissa G., Rossi A., Borrello M., Manzoni G.M., Mannarini S., Castelnuovo G., Molinari E. (2020). Development and Validation of a Self-Determination Theory-Based Measure of Motivation to Exercise and Diet in Children. Front. Psychol..

[96] Gearhardt A.N., White M.A., Masheb R.M., Morgan P.T., Crosby R.D., Grilo C.M. (2012). An examination of the food addiction construct in obese patients with binge eating disorder. Int. J. Eat. Disord..

[97] Di Giacomo E., Aliberti F., Pescatore F., Santorelli M., Pessina R., Placenti V., Colmegna F., Clerici M. (2022). Disentangling binge eating disorder and food addiction: A systematic review and meta-analysis. Eat. Weight Disord..

[98] Piccinni A., Bucchi R., Fini C., Vanelli F., Mauri M., Stallone T., Cavallo E.D., Claudio C. (2021). Food addiction and psychiatric comorbidities: A review of current evidence. Eat. Weight Disord..

[99] Maxwell A.L., Gardiner E., Loxton N.J. (2020). Investigating the relationship between reward sensitivity, impulsivity, and food addiction: A systematic review. Eur. Eat Disord. Rev..

[100] Carr M.M., Wiedemann A.A., Macdonald-Gagnon G., Potenza M.N. (2021). Impulsivity and compulsivity in binge eating disorder: A systematic review of behavioral studies. Prog. Neuropsychopharmacol. Biol. Psychiatry.

[101] Adamson S. (2017). Measuring Eating Compulsivity in the Wider Clinical Context. Subst. Use Misuse.

[102] Gearhardt A.N., White M.A., Masheb R.M., Grilo C.M. (2013). An examination of food addiction in a racially diverse sample of obese patients with binge eating disorder in primary care settings. Compr. Psychiatry.

[103] Castelnuovo G., Pietrabissa G., Cattivelli R., Manzoni G.M., Molinari E. (2016). Not Only Clinical Efficacy in Psychological Treatments: Clinical Psychology Must Promote Cost-Benefit, Cost-Effectiveness, and Cost-Utility Analysis. Front. Psychol..

